# Comparative Ultrastructure and Carbohydrate Composition of Gastroliths from Astacidae, Cambaridae and Parastacidae Freshwater Crayfish (Crustacea, Decapoda)

**DOI:** 10.3390/biom3010018

**Published:** 2012-12-21

**Authors:** Gilles Luquet, María S. Fernández, Aïcha Badou, Nathalie Guichard, Nathalie Le Roy, Marion Corneillat, Gérard Alcaraz, José L. Arias

**Affiliations:** 1 Biogéosciences, UMR 6282 CNRS-Université de Bourgogne, 21000 Dijon, France; E-Mails: nathalie.guichard@u-bourgogne.fr (N.G.); nathalie.le-roy@u-bourgogne.fr (N.L.R.); 2 Faculty of Veterinary and Animal Sciences, Universidad de Chile, Santiago, Chile; E-Mails: sofernan@uchile.cl (M.S.F.); jarias@uchile.cl (J.L.A.); 3 Biologie des Organismes et Ecosystèmes Aquatiques, UMR MNHN-CNRS 7208- UPMC-IRD 207, Station de Biologie Marine, 29900 Concarneau, France; E-Mail: aicha.badou@mnhn.fr (A.B.); 4 UPSP PROXISS, Département Agronomie Environnement, AgroSupDijon, 21000 Dijon, France; E-Mails: m.corneillat@agrosupdijon.fr (M.C.); g.alcaraz@agrosupdijon.fr (G.A.)

**Keywords:** biomineralization, calcification, calcium storage, carbohydrates, crayfish, Crustacea, gastrolith, organic matrix, proteoglycans

## Abstract

Crustaceans have to cyclically replace their rigid exoskeleton in order to grow.Most of them harden this skeleton by a calcification process. Some decapods (land crabs, lobsters and crayfish) elaborate calcium storage structures as a reservoir of calcium ions in their stomach wall, as so-called gastroliths. For a better understanding of the cyclic elaboration of these calcium deposits, we studied the ultrastructure of gastroliths from freshwater crayfish by using a combination of microscopic and physical techniques. Because sugars are also molecules putatively involved in the elaboration process of these biomineralizations, we also determined their carbohydrate composition. This study was performed in a comparative perspective on crayfish species belonging to the infra-order Astacidea (Decapoda, Malacostraca): three species from the Astacoidea superfamily and one species from the Parastacoidea superfamily. We observed that all the gastroliths exhibit a similar dense network of protein-chitin fibers, from macro- to nanoscale, within which calcium is precipitated as amorphous calcium carbonate. Nevertheless, they are not very similar at the molecular level, notably as regards their carbohydrate composition. Besides glucosamine, the basic carbohydrate component of chitin, we evidenced the presence of other sugars, some of which are species-specific like rhamnose and galacturonic acid whereas xylose and mannose could be linked to proteoglycan components.

## 1. Introduction

Arthropods possess a rigid exoskeleton, also called cuticle, which they have to replace regularly in order to grow. As a consequence, the life of these animals is closely linked to molting cycles. In most crustaceans, the cuticle is not only hardened by sclerotization, like in hexapods and chelicerates, but by calcification. Thereby, these animals have to find cyclically a source of calcium ions to mineralize each new exoskeleton [1,2,3,4,5]. The origin of the calcium used depends mainly on the way of life of the animal. For aquatic species, postmolt branchial calcium uptake largely contributes to the cuticle calcification, the food being to a lesser extent, another source of calcium ions. Terrestrial crustaceans (among them: amphipods, isopods and decapods), but also some aquatic decapod species, cyclically store calcium in their tissues. Thus, they have a reservoir of calcium ions immediately available after molting for quickly beginning to harden their cuticle in early postmolt. In this case, the complete mineralization of the new cuticle involves remobilization of the stored calcium, branchial uptake and food, the contribution of each source depending of the ecological niche of the animal. The storage organs, as well as the morphology of the storage structures, are very diverse. For example, terrestrial crabs, lobsters and crayfish store calcium in their stomach wall as one or two pairs of gastroliths. 

In Decapoda, the epithelial cells of the foregut are responsible for the synthesis of a cuticle covering the luminal surface. In crayfish, two gastroliths are elaborated during each premolt in the cardiac stomach wall in two anterior lateral specific discoid areas of the monolayered epithelium. Travis suggested that the gastroliths are elaborated between the epithelium and the cuticle [6,7]. She described the gastrolith structure of *Orconectes virilis* as “rounded, disc-shaped structures composed of an amorphous ground component and concentrically fibrous or filamentous lamellae”. 

It has been suggested for two freshwater crayfish species, *Procambarus clarkii* and *Cherax quadricarinatus* [8,9], that these calcium deposits are mainly constituted of amorphous calcium carbonate (ACC). It is notable that less than 10% of the calcium stored as gastroliths is used after ecdysis for the initial strengthening of some parts of the skeleton such as the gastric mill or the masticatory parts [1,6,7,10]. As for all the biologically-controlled mineralizations, the mineral is precipitated within an organic matrix network responsible for the initiation of this precipitation, the growth and the morphology of the calcification. The organic components are also thought to be involved in the choice of the calcium carbonate polymorph and finally in the stabilization over the time of the amorphous CaCO_3_ state (biogenic ACC), its purely mineral counterpart being the most unstable polymorph [11,12]. 

From these calcium storage structures, different categories of molecular components have been evidenced: calcium carbonate and to a lesser extent calcium phosphate for the mineral phase [10,13] and, for the organic phase, chitin, proteins, low-molecular weight components and proteoglycans. 

Four proteins directly extracted from gastrolith organic matrices have been well characterized and completely sequenced. The first one, named GAMP, was obtained from the crayfish *Procambarus clarkii* [14,15,16,17]. More recently, GAP 65 and GAP 10 were obtained from the Australian red claw crayfish, *Cherax quadricarinatus* [10,18,19]. Crustacyanin-A2 subunit, monomer of a hexadodecamer molecule responsible for astaxanthin binding, reveals to be also a component of *Cherax* gastrolith matrix [20]. Two others proteins have been identified but not yet sequenced, GAP 75 and Cq-CDA1 [13,21,22]. On the other hand, a previous work focusing on acid-soluble gastrolith matrix proteins extracted from crayfish of different genera had shown that if some polypeptides are probably common components of gastroliths whatever the genus considered, others seem specific [23]. 

Recently, Akiva-Tal *et al.* [24] and Sato *et al.* [25], by studying the cuticle and gastroliths of *Cherax quadricarinatus* and *Procambarus clarkii*, respectively, demonstrated the presence and suggested the involvement of phosphorylated energy-rich intermediates of the glycolytic pathway in the induction and stabilization of ACC. ACC particles would initially form due to an interaction of specialized matrix macromolecules bound to chitin in the CaCO_3_ precipitation process. Then, phosphoenolpyruvate (PEP) and 3-phosphoglycerate (3PI) would act by binding to the surface of ACC, thus inhibiting the transformation of ACC into a crystalline polymorph [25]. Citrate might be also involved in ACC stabilization by forming citrate-Ca^2+^-P complexes [24].

The presence and involvement of proteoglycans in crustacean biomineralizations had already been suggested [26,27]. Very recently, immunodetection of proteoglycans has been performed on histological sections of *Cherax quadricarinatus* stomach showing that four studied glycosaminoglycans (dermatan sulfate, keratan sulfate and chondroitin-4- and 6-sulfate) were detected both in the gastrolith-forming epithelium and in the gastrolith organic matrix [28].

Carbohydrates as components of biomineral organic matrices, other than chitin and glycosaminoglycans, have already been evidenced; however, only in calcifying phyla other than Arthropoda. For example, glycoproteins have been evidenced as intracrystalline components of the skeleton of coccolithophorids [29,30,31,32], cnidarians [33,34], brachiopods [35,36] and sea urchin embryos [37,38,39]. In mollusks, investigations have also been performed [40,41,42,43,44,45] evidencing glycoproteins or polysaccharides as components of matrix shell putatively involved in calcium carbonate crystallization (nucleation, crystal growth or crystal morphology). This family of carbohydrates (free or bound) could interact with growing CaCO_3_ crystals cooperatively with or independently of other interactions performed by members of another family of molecules such as highly acidic proteins [38,39,40,44]. *In vitro* experiments have been performed using matrix extracts, monosaccharides, trisaccharides or synthetic polymers of biological relevance for studying the involvement of saccharides or sulfate groups linked to polysaccharides in the CaCO_3_ crystal growth [32,38,45,46]. 

Using a combination of microscopy techniques (without or after different chemical treatments), XRD and FTIR analyses, we investigated in a comparative perspective the ultrastructure and carbohydrate composition of gastroliths from four crayfish species all belonging to the infra-order Astacidae (Crustacea, Decapoda, Malacostraca): three species from the Astacoidea superfamily, *Pacifastacus leniusculus* (Astacidae family), *Orconectes limosus* and *Procambarus clarkii* (Cambaridae family) and one species from the Parastacoidea superfamily, *Cherax quadricarinatus* (Parastacidae family). Furthermore, as regards the molecular differential composition of these gastroliths, we focused our interest on carbohydrates, performing analyses by high-performance anion exchange chromatography of the acid-insoluble gastrolith matrix from two Astacoidea and one Parastacoidea crayfish species.

## 2. Results and Discussion

### 2.1. Microscopic Analysis of the Gastrolith Structure

**Figure 1:**
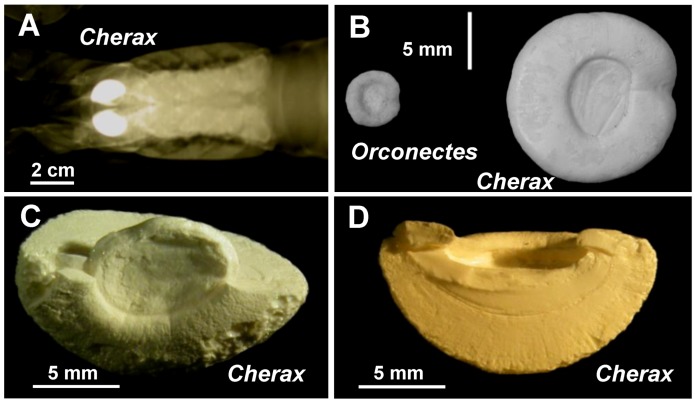
**A**: Radiography of the anterior part of a *Cherax quadricarinatus* specimen at the end of the calcium storage process, which occurs all during the premolt, showing the localization of two X-ray dense gastroliths. **B**: Gastroliths (light microscopy) from *Orconectes limosus*, at left, and from *Cherax quadricarinatus*, at right. The macrostructural aspect is similar. The difference of size is proportional to the size of adult specimens of the two species. The flat or slightly concave side on the stomach lumen side is shown. **C–D**: Broken gastroliths from *Cherax quadricarinatus* showing the semi-spherical and internal aspect of a gastrolith. **C**: the flat disc visible in the center corresponds to the oldest part of the gastrolith, *i.e.* the beginning of the calcium storage. **D**: The homogeneous dense and striated internal structure is well visible on this cross-section.

**Figure 2:**
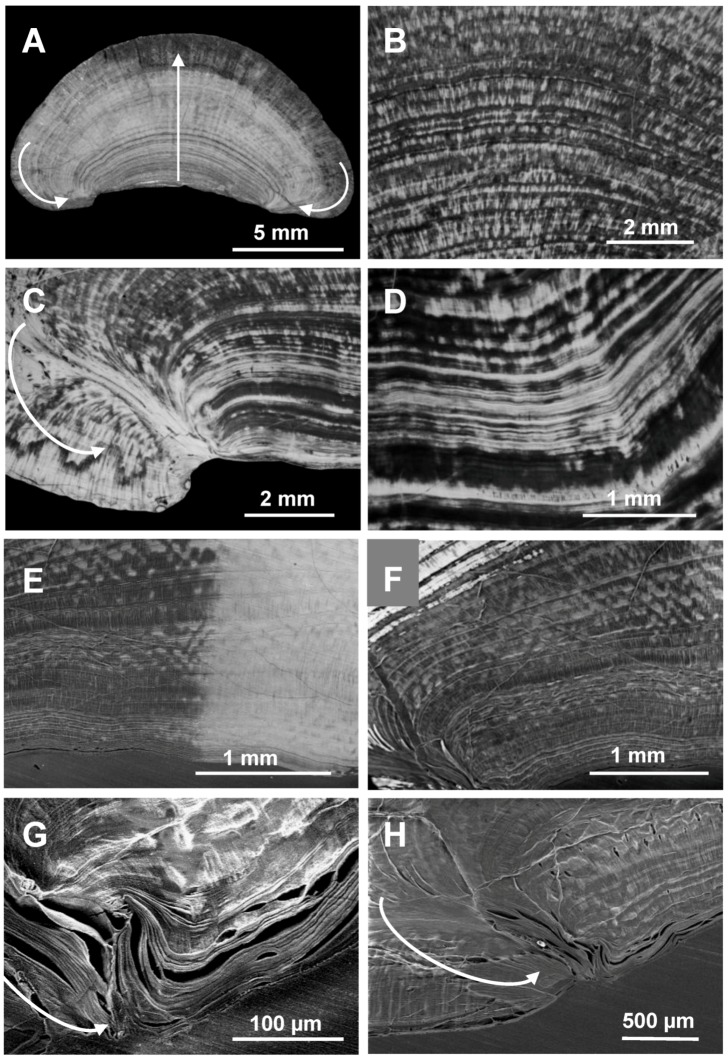
Cross-section of a *Cherax quadricarinatus* gastrolith showing an irregular layered structure. **A–D**: Light microscopy, without any treatment. A: cross-section of a whole gastrolith. The central arrow indicates the growing direction, the lateral arrows indicate the lateral covering process occurring at the end of the storage in late premolt. **B–D**: magnification of different areas of a cross-section. Dense calcium carbonate layers of different widths alternate with less dense layers. **E–H**: (E–F: Light microscopy, G–H: SEM) the presence and direction of organic matrix layers are clearly shown after partial surface decalcification (grey areas; the bright areas correspond to the native structure).

In crayfish, two gastroliths are elaborated during each premolt in the cardiac stomach wall [7]. When fully developed, at ecdysis (Figure 1A), all the gastroliths exhibit the same general morphology. They are semi-spherical, the face on the stomach lumen side (the first synthesized part of the gastrolith) appears flat or slightly concave whereas the other face, in close contact with the calcifying epithelium, is convex (Figure 1B–D; Figure 2A).

Gastrolith cross-sections observed in light microscopy show a dense structure of successive calcified layers of irregular width (Figure 2A–F). By different treatments with acetic acid, we obtained more or less partially decalcified gastroliths. After slight decalcification (Figure 2E–H), the organic matrix is revealed, showing that the layers are parallel to each other and to the cuticle at the beginning of the storage process, then they become concentric, appearing more and more enlarged in correspondence with the regular (almost linear) calcium precipitation occurring in late premolt until ecdysis [9]. Finally, at the end of the process, they develop considerably, covering laterally the first formed layers (Figure 2A, 2G and 2H, arrows). Organic layers are clearly visible when decalcification is a little bit stronger (Figure 2F).

**Figure 3:**
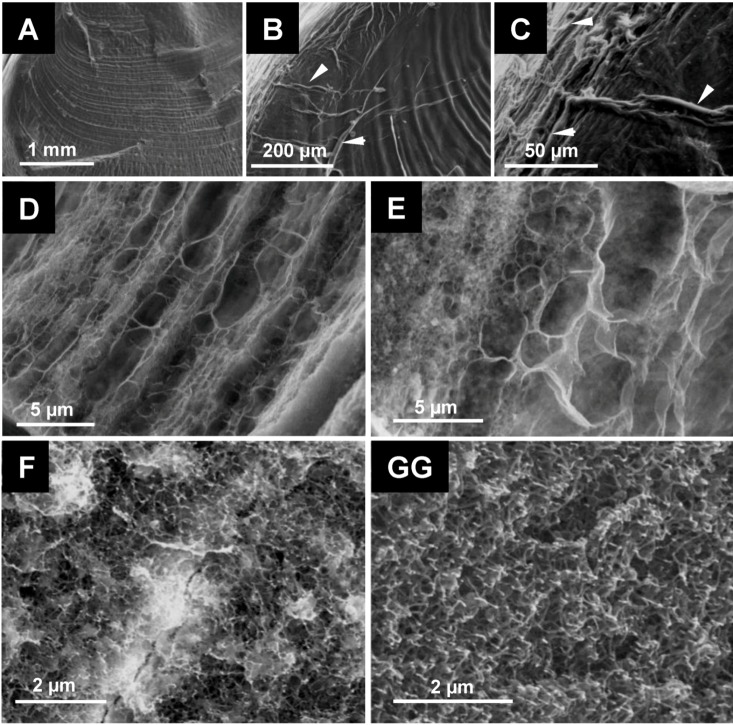
Structural aspect of the organic matrix of *Cherax quadricarinatus* gastrolith (SEM). **A–C**: slight surface decalcification showing the striated aspect of the gastrolith (A) and at higher magnification the presence of large fibers of organic matter (B and C; arrows heads). **D–E**: organic matrix fibers forming parallel layers of different widths and densities observed after surface treatment by acetic acid (partial surface decalcification). Transverse fibers are also visible resulting in a pocket-like structure. **F–G**: high magnifications of regions exhibiting a dense network of organic nanofibers, subdividing the previously revealed pocket-like compartments in micro-pockets.

After a few minutes incubation of broken gastroliths in 5% acetic acid, the large-diameter organic fibers, corresponding to chitin-protein fibers, are well visible on surface (Figure 3A–C). When more decalcified, a complex network of fibers appear showing not only the parallel organic layers previously observed in light microscopy but also cross fibers of similar size (Figure 3D and 3E). This transversal striated structure was already visible in some pictures of Figure 2 (notably in Figure 2A). 

All these fibers organize a 3-dimensional framework forming pockets of different sizes. These pockets are themselves subdivided by fibers of nanometric diameters resulting in nano-compartments filled with CaCO_3 _(Figure 3F and Figure 3G).

**Figure 4:**
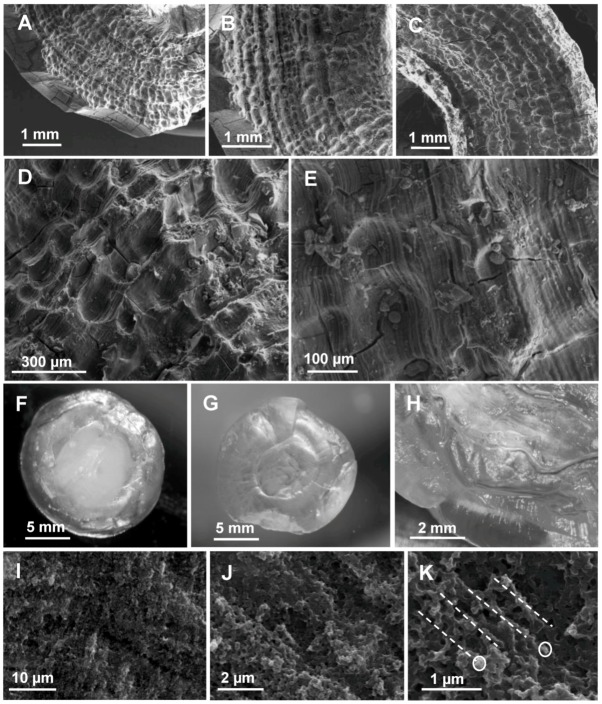
**A–E**: SEM observations of the external aspect of a *Cherax* gastrolith in postmolt after a beginning of natural digestion in the stomach (different magnifications). A layered pocket-like structure is observed. **F–H**: Sponge-like aspect of a *Cherax* gastrolith after a slow decalcification by 2.5% acetic acid (Light microscopy). F: after two days treatment, the center of the gastrolith (chalky) is not completely decalcified, G: Completely decalcified gastrolith after one week treatment (translucent aspect). H: Cross-section of a fully decalcified gastrolith. **I–K**: Internal aspect observed after fracture by SEM (three magnifications of the same sample are shown). The layered aspect is visible; each layer is formed by alignments (dashed lines) of dense aggregated spherical nanoparticles (circles).

Such a pocket-like structure is visible on gastroliths extracted from the stomach of animals in early postmolt, which means after the beginning of a natural decalcification (Figure 4A–E). The decalcification process seems to occur “pocket by pocket” following enzymatic and/or acidic digestion within the stomach.

After extraction, lyophilisation and weighing of the IM and SM fractions, the whole organic matrix was estimated to represent between 3% to 5%, w:w, of a whole gastrolith. The organization and quantitative importance of the matrix network in the general morphology of a gastrolith is in accordance with the sponge-like structure obtained after complete decalcification of a gastrolith. After such treatment, if the volume is reduced because calcium carbonate has been discarded, the general morphology is conserved (Figure 4F–H) in accordance with the presence of a very dense and organized organic network.

When observed in light microscopy or in SEM, the layers appear densely and homogeneously calcified. At higher magnification, the precipitated mineral is present as layers of small spherules (about 100 nm diameter; Figure 4I–K), putatively aligned along and around organic matrix fibers as demonstrated for the outer decapod cuticle [47,48].

### 2.2. Mineralogical Composition

The determination of the mineralogical composition of gastroliths was performed by FTIR and XRD analyses. The CaCO_3_ FTIR spectrum is characterized by four infrared vibration modes of the free CO_3_^2−^ ions at about 1060–1080 cm^−1^ (ν_1_), 870–860 cm^−1^ (ν_2_), 1390–1430 cm^−1^ (ν_3_) and 680–710 cm^−1^ (ν_4_) [49]. The precise absorption bands of each vibration vary depending of the polymorph of CaCO_3_. FTIR ACC spectrum is characterized by a slight peak at about 860 cm^−1^ (ν_2_), a broad/double peak at 1000–1070 cm^−1^ (ν_1_) and a double peak at 1380–1470 cm^−1^ (ν_3_). 

As for the putative presence of phosphate compounds, the PO_4_^3−^ ions infrared vibration modes give rise to two characteristic absorption bands located at 1200–900 cm^−1^ and 650–550 cm^−1^[49].

**Figure 5:**
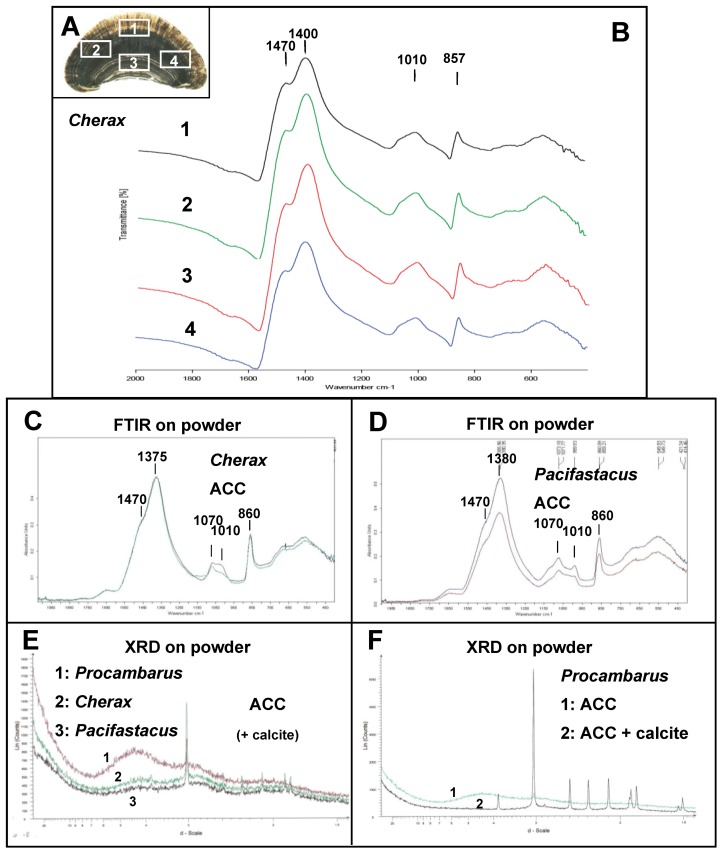
Analysis of the gastrolith CaCO_3_ polymorph by Fourier transform infrared (FTIR) and XRD. **A**: Cross-section of a gastrolith from *Cherax* showing four different locations where FTIR analyses have been performed. **B**: FTIR spectra obtained from the 4 gastrolith areas shown in A. The four spectra are similar and are typical of the ACC polymorph. **C–D**: ACC FTIR spectra obtained on gastrolith powder from *Cherax quadricarinatus* (C) and *Pacifastacus leniusculus* (D). **E–F**: XRD spectra obtained on gastrolith powder (E) from three different species of crayfish and (F) from *Procambarus clarkii* (1. ACC polymorph profile, 2. ACC + calcite profile).

When performed on a *Cherax* gastrolith cross-section in four different sites, the obtained FTIR spectra demonstrate everywhere the presence of ACC (Figure 5A and B). Similarly, when performed on powdered gastrolith from *Cherax quadricarinatus* (Figure 5C) or *Pacifasctacus leniusculus* (Figure 5D), FTIR spectra are characteristic of ACC.

**Figure 6:**
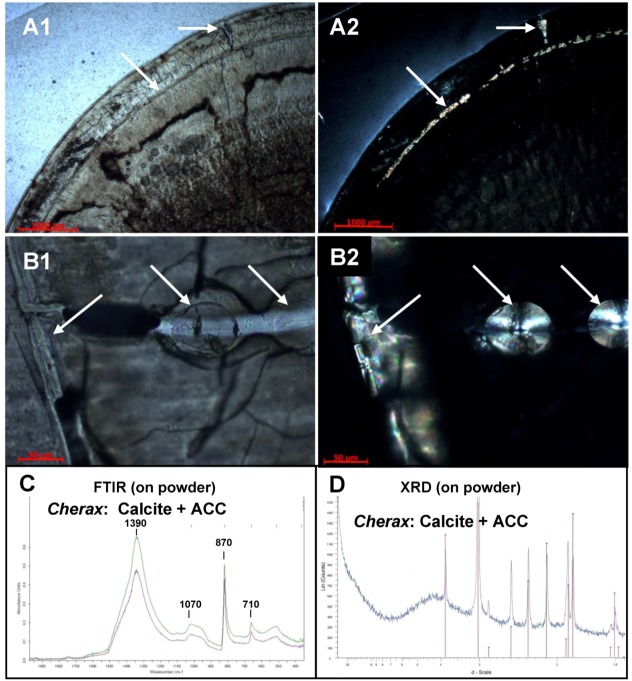
Observation of cross-sections of *Cherax quadricarinatus* gastrolith by **A1**, **B1**: Non-Polarized Light (NPL) microscopy and **A2**, **B2**: Polarized Light microscopy (PLM). Most of the gastrolith appears completely dark in PLM suggesting a non-crystallized structure. In a few samples, some areas exhibit a birefringent aspect (arrows) corresponding to the presence of crystallized mineral. In these particular cases, FTIR (**C**) and XRD (**D**) spectra show a mix of two CaCO_3_ polymorphs: ACC and calcite.

For comparison, calcite spectrum is characterized by a small peak at 710 cm^−1^, a sharp and high peak at 870 cm^−1^ and a single peak at 1390 cm^−1^ (see Figure 6C) [50]. On the other hand, the FTIR spectrum obtained is neither characteristic of aragonite nor characteristic of another biogenic crystalline calcium carbonate polymorph, which have been analyzed previously [50,51,52].

We do not exclude the possibility that the absorption band visible at 1070 cm^−1^ could be also related to the presence of PO_4_^3−^ ions as indicated for the analysis of the giant prawn exoskeleton [49]. Nevertheless, no absorption band is visible in the range 650–550 cm^−1^.

XRD spectra obtained on gastrolith powder exhibit a typical ACC spectrum with no narrow sharp peaks but a very broad band spreading from 700 to 330° (2-Theta scale) [53,54]. It is to notice that a typical ACP spectrum exhibits similarly two diffuse broad bands as visible for example on the spectrum 1 of Figure 5E.

In some analyses, some peaks, more or less high, can be referred to calcite (Figure 5E). Figure 5F shows two XRD spectra obtained from gastroliths of the same crayfish species. One is typical of ACC whereas the other exhibits a mix of ACC and calcite. The presence of a natural mix of amorphous and crystallline CaCO_3_ has previously been reported [49,55].

Another explanation has been obtained from observations of gastrolith cross-sections in polarized light microscopy. Figure 6 shows pictures obtained in bright-field light microscopy (Figure 6A1 and B1) and polarized light microscopy (Figure 6A2 and B2) on gastrolith cross-sections. The surface of the gastrolith appears everywhere opaque except for birefringent areas corresponding to crystalline part (Figure 6A2 and 6B2, arrows). Light microscopy showed that these zones are connected to the surface (Figure 6A1 and 6B1, arrows) and correspond to artificial fractures within which aqueous solution penetrated the gastrolith. This produced a local solubilization of ACC followed by a secondary recrystallization in calcite, the most stable CaCO_3_ polymorph, as demonstrated by XRD and FTIR spectroscopy (Figure 6C and 6D). Such an experimental transformation may occur during the extraction procedure or during the NaOCl washing step, which has the purpose of eliminating putative organic contaminants (originating from the stomach epithelium for example). A similar experimental secondary crystallization has been previously observed for ACC storage deposits elaborated by a terrestrial amphipod, *Orchestia cavimana* [56].

### 2.3. Sugar Analysis and FTIR Matrix Analysis

Among the molecules of the organic matrix of crayfish gastrolith that are considered as mainly composed of chitin-protein fibers, proteins and proteoglycans have received the most attention so far [13,14,15,16,17,18,19,20,21,22,23,24,25,28].

In this work, we performed an analysis of monosaccharides from the acetic acid-insoluble fraction of matrix gastrolith (IM; obtained as described in the Experimental section) originating from three crayfish species by high performance anion-exchange chromatography after TFA hydrolysis.

The amounts of the detected monosaccharides are reported in Table 1A and classified by their order of abundance in *Cherax quadricarinatus*. 

**Figure 7:**
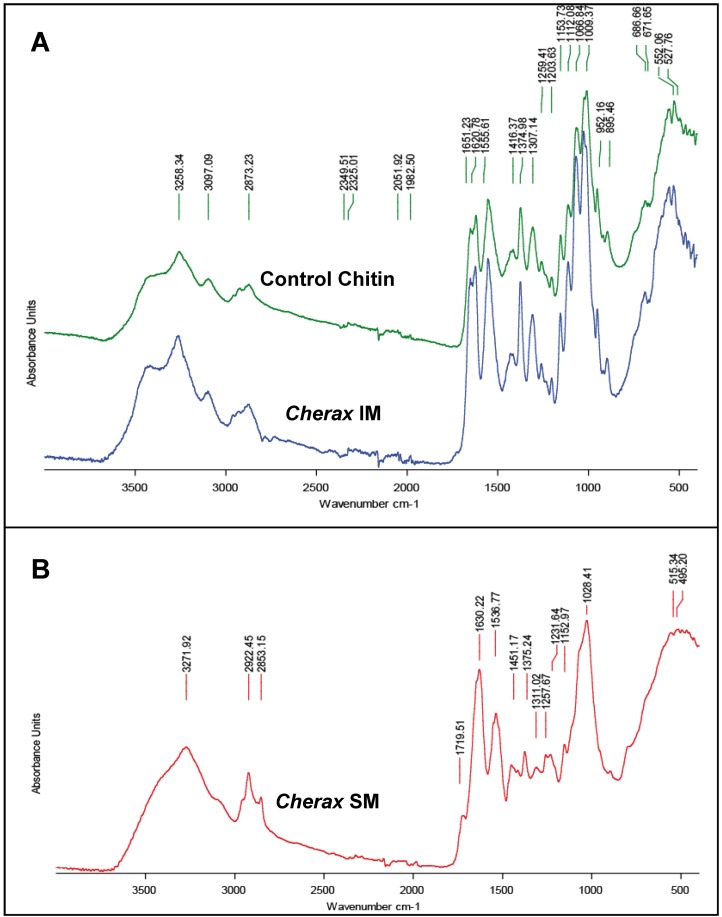
FTIR spectra of organic matrix fractions of gastrolith from *Cherax quadricarinatus*. **A**: Spectrum of the acid acetic-insoluble fraction (IM). This spectrum is similar to a control king crab chitin spectrum. **B**: Spectrum of the acid acetic-soluble fraction (SM).

For all the analyzed crayfish species, the IM matrix exhibits a high level of glucosamine, from about 65 to 72.5% of the total sugar content (Table 1C), in accordance to the presence of large chitin fibers, which cannot be solubilized and depolymerized during the acetic acid decalcification process, nor during the NaOH treatment. The high level of glucosamine in the IM matrix is in accordance with the FTIR spectrum obtained from the *C. quadricarinatus* IM fraction (Figure 7A). This spectrum is highly similar to the spectrum obtained by using commercial crustacean chitin (from the king crab; Sigma™), whereas the soluble matrix FTIR spectrum (Figure 7B) obtained from *C. quadricarinatus* is a little bit different, even though some absorption peaks could be attributed also to the presence of chitin. Notably, if the glucosamine level is about 72.4% for *Cherax* in the IM fraction (Table 1C), it remains not negligible in the SM fraction (about 35.8%) [28]. This could be due to the presence of very small chitin fibers (see Figure 3F and G) subdividing the large pocket-like compartments into micro-units framed by larger chitin fibers. Some of these nanometric diameter fibers could be depolymerized and released during the decalcification process, remaining in the supernatant after centrifugation of the decalcifying solution.

Besides glucosamine, we detected other monosaccharides, each one representing less that 10% of the total sugar content of the IM matrix (Table 1B). The order of abundance of these other sugars is not exactly the same from one crayfish to another even though xylose, arabinose, glucose and mannose are always the most abundant (from about 3 to 9.5%; Table 1C). It is notable that glucuronic acid was not detected in *C. quadricarinatus*, Parastacidae, and that galacturonic acid and rhamnose were only found in *O. limosus*, Cambaridae.

The presence of the poorly abundant monosaccharides cannot be really explained in all cases, for example in the case of arabinose, glucose and fucose. They could be free or part of glycosyl groups linked to proteins, the precise function of which remains to be determined.

Nevertheless, the presence of xylose and mannose may alternatively be related to the presence of proteoglycans [28]. Indeed, chondroitin sulfate, dermatan sulfate and keratan sulfate are proteoglycans composed of a glycosaminoglycan (GAG) bound to a protein core via a xylose sugar. On the other hand, keratan sulfate II (KSII) is composed of a glycosaminoglycan (GAG) bound to a protein core via a mannose sugar [57,58].

How the biogenic ACC polymorph can be induced and stabilized over time is a long-standing and disputable question that has received recent attention. Stabilization could be due to stabilizing molecules or to molecules able to inhibit the transformation of ACC to another more stable crystalline polymorph. It was suggested that specialized macromolecules (acidic proteins, phosphoproteins, sulfated glycoproteins, polysaccharides) or ions such as magnesium or phosphate could contribute to the ACC determination/stabilization [18,20,46,59,60,61,62,63,64,65,66,67]. 

**Table 1:** Relative distribution of monosaccharides in the acid insoluble gastrolith organic matrix from three crayfish species determined by high performance anion-exchange chromatography after trifluoracetic acid (TFA) hydrolysis. **A**. Diagram representation of the monosaccharides distribution in *Cherax quadricarinatus* (blue), *Pacifastacus leniusculus* (red), *Orconectes limosus*, (green). **B**. Enhanced diagram representation of the minor monosaccharides (scale of the y-axis: from 0 to 10%). **C**. Amount of the main monosaccharides (expressed in % total sugar; mean ± sd from three analyses).

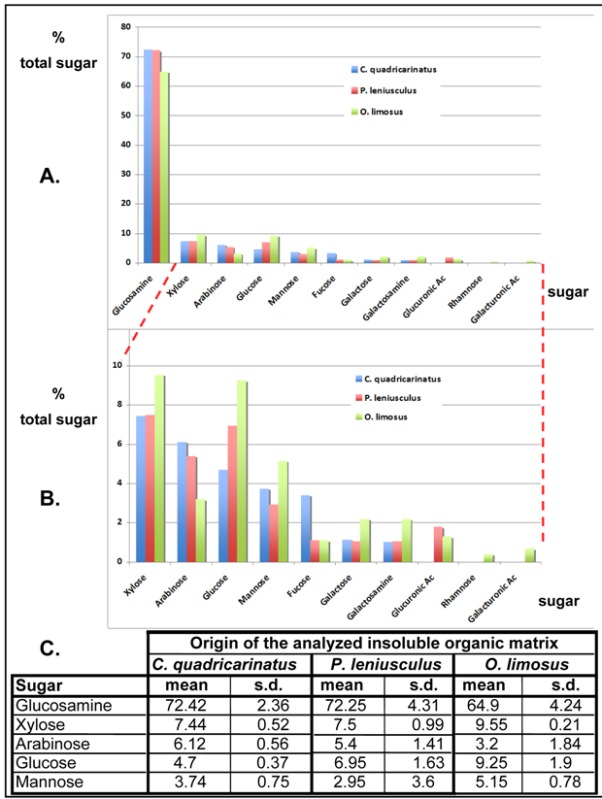

The gastrolith matrix protein GAP 65, with putative phosphorylation sites, was shown to *in vitro* inhibit calcium carbonate crystallisation [18]. More recently, Bentov *et al.* [13] demonstrated the ability of an isoluble matrix fraction to *in vitro* induce ACC. This ability was attributed to a doublet of phosphorylated and calcium-binding polypeptides migrating at about 70-75 kDa in SDS-PAGE. Nevertheless, these proteins were not purified and tested alone for their capacity to induce ACC. 

In gastroliths, as putative molecules bound to chitin involved in the ACC induction process upstream the ACC stabilization effect of low-molecular weight components [24,25], we may suspect proteins with post-translational modifications such as phosphorylations, sulfations or glycosylations. Proteoglycans can also be considered as another category of gastrolith matrix components, bound or not to chitin, putatively involved in the ACC determination.

## 3. Experimental Section

### 3.1. Animals

The Australian red claw crayfish, *Cherax quadricarinatus*, were bred in a farm located on the campus of the University of Chile, Santiago de Chile. Gastroliths were extracted from animals at ecdysis (moment when the animals shed their old cuticle). The American crayfish, *Orconectes limosus*, and the Californian crayfish, *Pacifasctacus leniusculus*, were fished in the Parc du Morvan and in Burgundy rivers, France, then kept in freshwater tanks in the University of Burgundy until ecdysis. Gastroliths of the Louisianan crayfish, *Procambarus clarkii*, were obtained from animals originating from the region of Bordeaux, France, and were a gift from Dr. J.P. Delbecque from the “Centre de Neurosciences Intégratives et Cognitives”, University of Bordeaux, France. 

### 3.2. Samples Preparation and Chemical Treatments

Gastroliths were immersed in 10% (v/v) NaOCl overnight at 4 °C under gentle stirring to remove superficial organic contaminants then thoroughly rinsed with distilled water and air-dried at 37 °C for 1 hour.

For XRD or IR spectroscopy analyses, gastroliths were ground to a powder and kept at room temperature and dry air until used. 

For sugar and matrix analyses, powder was decalcified during 48 h in cold acetic acid (10% v/v) at 4 °C. The solution was then centrifuged 30 min at 3893 g at 4 °C. The pellet, corresponding to the acetic acid-insoluble matrix (IM), was rinsed 10x with 50 ml MilliQ water (Millipore^®^), freeze-dried and weighed. The supernatant containing the acetic acid-soluble matrix (SM) was filtered (5 µm cut-off) and concentrated with an Amicon ultrafiltration system on a Millipore^®^ membrane (YM10; 10-kDa cut-off). The solution (approximately 5 mL) was extensively dialyzed against MilliQ water (3 days, several water changes) before being freeze-dried and weighed.

For chemical treatment, pieces of freshly broken gastroliths were incubated in acetic acid 10% or 5% (30 sec), depending of the level of decalcification expected for examining the structure on the surface or deeper. Then, they were carefully rinsed with distilled water and air-dried before microscopic observations. Full gastroliths were completely and slowly decalcified with 2.5% acetic acid to avoid desorganizing the organic network.

### 3.3. Light Microscopy, Scanning Electron Microscopy and Polarized Light Microscopy

For light microscopy observations, gastroliths were embedded in araldite and cross-sections of gastroliths (50-µm thickness) were performed. They were observed using a Nikon Eclipse E200 microscope or a Nikon SMZ800 binocular stereomicroscope.

For SEM observations, gastroliths were slightly broken in a mortar with a pestle. Some pieces were observed without any further treatment, others were decalcified with 1M EDTA for 2 min for a slight surface treatment. For a more intensive decalcification, samples were incubated 30 min in 10% acetic acid. After chemical treatment, samples were air-dried and carbon covered. They were observed using a Hitachi TM-100 scanning electron microscope or a JEOL 6400 F scanning electron microscope (for high magnifications).

Observations in polarized light were performed using a Zeiss Axiophot microscope. 

### 3.4. X-ray Diffraction

X-ray diffraction spectra were obtained from powdered gastroliths using a Siemens Buker D500 X-ray diffractometer. CaCO_3_ calcite (calcite D, ref 00-024-0027, Siemens) peaks were used as a reference to determine the amorphous to calcite transformation. 

### 3.5. Fourier transform infrared spectroscopy

Infrared spectroscopy was performed on powdered samples by direct ATR transmission according to the method previously described [52,68]. IR absorption spectra were obtained using a Fourier transform infrared (FTIR) spectrometer (Bruker-Vector 22) in the range 2000–200 cm^−1^, 2 cm^−1^ resolution and accumulation of 32 scans.

### 3.6. Sugar Analysis

For sugar analysis, we used lyophilized IM matrix samples from 3 different species. Each of these samples corresponds to a pool of six gastroliths ground in powder and decalcified as described above (see *3.2*). The lyophilized samples were hydrolyzed in trifluoroacetic acid (TFA, 2 M) at 105 °C for 4 h. Samples were evaporated to dryness before being resuspended with NaOH 20 mM. The acidic, neutral, and amino sugar contents of the hydrolysates were determined by high-performance anion exchange-pulsed amperometric detection (HPAE-PAD) by using a CarboPac PA100 column (Dionex P/N043055), according to the Dionex instructions. Non-hydrolyzed samples were analyzed similarly, in order to detect free monosaccharides in the samples. The results obtained (means±standard deviations) for each species come from three analyses performed with three different pools of gastroliths. Blank experiments were performed using BSA diluted in 10% acetic acid and submitted to all the steps of the extraction procedure. It is to notice that this technique does not allow the quantification of sialic acids, which are destroyed during hydrolysis with TFA, nor the detection of iduronic acid because of the lack of available standards. All the analyses have been performed in the same lab, UPSP PROXISS, AgroSupDijon, Dijon, and by the same person to avoid possible experimental discrepancy.

## 4. Conclusions

In this work, we observed that, for all the crayfish species considered, the gastroliths are mainly composed of ACC, in accordance with the aim of the cyclic elaboration of these calcifications: the transitory storage of calcium ions [58]. We evidenced that their structures are identical from the macroscopic to the microscopic level presenting a similar three-dimensional framework of chitin-protein fibers. Furthermore, we observed a complex organization of abundant fibers at the nanoscale. concentrically and transversally distributed, forming pockets of different sizes within ACC is precipitated. Nevertheless, gastroliths, depending of the species of origin, are not completely similar at the molecular level, as shown in this work for the sugar composition, and are probably built with a different cocktail of components among which proteins, putatively post-translationally modified, proteoglycans and sugars, free or bound to a protein core and probably also modified (sulfated notably). Some of these components, linked to chitin, which remain to be precisely characterized, are probably involved in the precipitation of CaCO_3_. Then other matrix molecules, putatively of lower molecular weight, would act to stabilize these reservoirs of calcium ions over time under the amorphous form of calcium carbonate.
